# Distinct implications of body mass index in different subgroups of nonobese patients with heart failure with preserved ejection fraction: a latent class analysis of data from the TOPCAT trial

**DOI:** 10.1186/s12916-022-02626-4

**Published:** 2022-11-02

**Authors:** Bin Dong, Yiling Yao, Ruicong Xue, Weihao Liang, Jiangui He, Fangfei Wei, Yugang Dong, Xin He, Chen Liu

**Affiliations:** 1 Department of Cardiology, The First Affiliated Hospital of Sun Yat-sen University, 58 Zhongshan 2nd Road, Guangzhou, 510080 China; 2 NHC Key Laboratory of Assisted Circulation (Sun Yat-sen University), Guangzhou, China; 3 National - Guangdong Joint Engineering Laboratory for Diagnosis and Treatment of Vascular Diseases, Guangzhou, China; 4 Department of Cardiology, the Affiliated Sanming First Hospital of Fujian Medical University, Sanming, China

**Keywords:** HFpEF, Obesity paradox, Nonobese HFpEF, Latent class analysis

## Abstract

**Background:**

Obesity is a well-defined risk factor for heart failure with preserved ejection fraction (HFpEF), but it is associated with a better prognosis in patients with diagnosed HFpEF. The paradoxically poor prognosis in nonobese patients with HFpEF may be driven by a subset of high-risk patients, which suggests that the nonobese HFpEF subpopulation is heterogeneous.

**Methods:**

Latent class analysis (LCA) was adopted to identify the potential subgroups of 623 nonobese patients enrolled in the TOPCAT trial. The baseline characteristics of the identified nonobese subgroups were compared with each other and with the obese patients. The risks of all-cause, cardiovascular, and noncardiovascular mortality, and an HF composite outcome were also compared.

**Results:**

Two subgroups of nonobese patients with HFpEF (the physiological non-obesity and the pathological non-obesity) were identified. The obese patients were younger than both nonobese subgroups. The clinical profile of patients with pathological non-obesity was poorer than that of patients with physiological non-obesity. They had more comorbidities, more severe HF, poorer quality of life, and lower levels of physical activity. Patients with pathological non-obesity showed low serum hemoglobin and albumin levels. After 2 years of follow-up, more patients in the pathological group lost ≥ 10% of body weight compared with those in the physiological group (11.34% vs. 4.19%, *P* = 0.009). The prognostic implications of the two subgroups were opposite. Compared to patients with obesity, patients with physiological non-obesity had a 47% decrease in the risk of HF composite outcome (hazard ratio [HR] 0.53, 95% confidence interval [CI] 0.40–0.70, *P*<0.001) and a trend of decreased all-cause mortality risk (HR 0.75, 95% CI 0.55–1.01, *P*=0.06), while patients with pathological non-obesity had a 59% increase (HR 1.59, 95% CI 1.24–2.02, *P*<0.001) in all-cause mortality risk.

**Conclusions:**

Two subgroups of nonobese patients with HFpEF with distinct clinical profiles and prognostic implications were identified. The low BMI was likely physiological in one group but pathological in the other group. Using a data-driven approach, our study provided an alternative explanation for the “obesity paradox” that the poor prognosis of nonobese patients with HFpEF was driven by a pathological subgroup.

**Supplementary Information:**

The online version contains supplementary material available at 10.1186/s12916-022-02626-4.

## Background

Obesity is a growing public health problem worldwide. Although obesity is a well-defined risk factor for numerous cardiovascular diseases [1], a high body mass index (BMI) is associated with better prognosis in patients with diagnosed heart failure (HF), which is known as “obesity paradox” [2–4]. For the past decade, multiple theories have been put forward to explain this phenomenon. Some researchers think that the biological effect of adiposity mainly depended on fat distribution [5]. The difference in fat distribution in obese patients might confound the obesity-prognosis association. Others believe that some patients with HF suffered from wasting syndrome and became nonobese due to unintentional weight loss, which accounts for the poor prognosis in nonobese patients with HF [6]. Most of the theories are supported by evidence, but none of them are conclusive.

Among patients with HF with preserved ejection fraction (HFpEF), obesity is highly prevalent and considered a fundamental pathogenic factor [7]. Obese HFpEF is currently considered a distinct phenotype of HFpEF [8, 9]. In a cross-sectional study that compared the characteristics of obese vs. nonobese HFpEF, the obese patients had more concentric left ventricular remodeling, right ventricular dysfunction, more adverse hemodynamics, and worse exercise capacity [8]. These data suggested that obesity did have a negative effect on the heart of patients with HFpEF. However, other studies have shown that nonobese patients as a whole had a higher risk of adverse outcome [2, 10, 11]. These inconsistent results led to a hypothesis that nonobese patients with HFpEF might represent a heterogeneous group of patients. Given that at least a part of the nonobese patients with HFpEF should represent a relatively “healthy” condition free from obesity-related cardiac damage, the poor prognosis of the overall nonobese population might be driven by a high-risk subgroup.

Latent class analysis (LCA) is one of the clustering techniques which aims to identify potential subgroups of individuals who share similar characteristics [12]. It is a finite mixture model that assumes the existence of unobserved subgroups within an overall population. Individuals within a subgroup share a similar pattern of analyzed variables, while patterns between the subgroups are different from each other [12]. Unlike other clustering methods that only handle continuous variables, LCA can be used to analyze different types of variables. LCA has been used to explore underlying subgroups of multiple diseases [13–15].

Therefore, we adopted the LCA technique for nonobese (BMI < 30 kg/m^2^) patients with HFpEF from Treatment of Preserved Cardiac Function Heart Failure with an Aldosterone Antagonist (TOPCAT) trial to explore potential subgroups of nonobese patients with HFpEF.

## Methods

### Patients

This is a post hoc analysis of the TOPCAT trial. We obtained the data from the Biologic Specimen and Data Repository Information Coordinating Center of the US National Heart, Lung, and Blood Institute via an approved proposal. TOPCAT was a multicenter, international, randomized, double-blinded placebo-controlled trial, which enrolled 3,445 adults with HFpEF from over 200 clinical centers. The aim of the trial was to investigate whether spironolactone could improve prognosis in patients with HFpEF. The design of TOPCAT has been published in detail previously [16]. Briefly, eligible patients were required to have: an age of ≥ 50 years; at least one symptom and one sign of heart failure; left ventricular ejection fraction of ≥ 45%; controlled systolic blood pressure; serum potassium of < 5.0 mmol/L; elevated natriuretic peptide levels within the last 60 days or at least one HF hospitalization in the last 12 months. The trial was approved by the ethics committee at each study center. Every participant signed an informed consent form.

In our study, we excluded patients enrolled from Russia and Georgia (*n* = 1678), for the regional difference in TOPCAT [17]. Patients with missing data on height or weight (*n* = 9) were also excluded. Finally, a total of 1758 patients were analyzed in our study.

### Phenotyping using LCA

Obese and nonobese patients were defined by a BMI cutoff value of 30 kg/m^2^, and LCA was performed among the nonobese patients (*n* = 623). For the LCA variables, two steps of selection were performed. The first step was manual selection. The inclusion criteria in this step were not stringent to avoid subjective bias and missing unrecognized clustering variables. Variables about demographic features, medical history, physical examination, and laboratory investigation potentially related to HFpEF prognosis or pathophysiology were eligible. Two junior cardiologists (BD and XH) screened the variable dictionary of the TOPCAT dataset, and potential candidates were adjudicated by a senior cardiologist (CL). Thirty-six variables were manually selected and are listed in Additional file 1: Table S1. The second selection step was algorithm-based. A backward stepwise algorithm was adopted to discard redundant and noninformative variables because including these variables in the LCA model will negatively affect the clustering performance [18]. In brief, all of the 36 variables were included in the LCA model. The algorithm assessed the usefulness of each variable and discarded the least useful one at each step. To avoid missing potential clustering variables, the algorithm reevaluated the usefulness of all discarded variables after each removal and tried to add the most likely useful one back to the model. The action of “removal” or “adding” would be accepted or rejected depending on whether it improved the performance of the model assessed by the difference in the Bayesian Information Criterion (BIC). This calculation iterated until no further action was accepted. The abovementioned selection was achieved using the *LCAvarsel* package in R. The process of algorithm-based variable selection is presented in Additional file 1: Table S2. Finally, 10 variables namely race, Kansas City Cardiomyopathy Questionnaire (KCCQ) overall summary score, previous HF hospitalization, diabetes, diastolic blood pressure, diuretic usage, beta-blocker usage, hemoglobin, albumin and glomerular filtration rate (GFR) were selected for the LCA model (Additional file 1: Table S3). The optimal number of subgroups was determined by comprehensively evaluating BIC, Akaike information criterion (AIC), G^2^, *χ*^2^, adjusted BIC (aBIC), and consistent AIC (cAIC). Three out of the six indexes supported the optimal group number to be two (Additional file 1: Table S4). Partial probabilities of the phenotype membership for the selected variables are summarized in Additional file 1: Table S5. The probability of a patient belonging to a certain group was determined by multiplying partial probability of each variable. Finally, the patient was assigned to the group that had the highest probability.

### Outcome of interest

The outcomes in our study included all-cause mortality, cardiovascular mortality, and a composite outcome of cardiovascular mortality, aborted cardiac arrest, or HF hospitalization defined as primary by TOPCAT. Noncardiovascular causes accounted for a larger proportion of mortality in HFpEF compared with that in heart failure with reduced ejection fraction [19, 20]. Therefore, noncardiovascular mortality was also evaluated. All the outcomes were adjudicated by a clinical end-point committee at Brigham and Women’s Hospital.

### Statistical analysis

Comparisons were made between the two nonobese groups and the obese group. The continuous variables are presented as mean ± standard deviation or median with the interquartile range depending on their normality. The categorical variables were described using frequencies with percentages. Differences in baseline characteristic data among the three groups were compared using a one-way ANOVA or Kruskal-Wallis test for continuous variables. Bonferroni’s test was applied for pairwise comparisons in the post hoc analysis if the results were significant. The categorical variables were compared using chi-squared test. The Kaplan-Meier survival curve with a log-rank test was used to show the differences in survival among the groups. Cox proportional-hazards regression model was performed to evaluate the association of different phenotypes with all-cause mortality and the composite outcome, and the obesity group served as the reference group. For cardiovascular and noncardiovascular mortality, Fine and Grey’s competing risk regression model was used to adjust for the competing risk from each other.

To evaluate whether the prognostic implications of the nonobese subgroups were independent of demographic differences and randomized treatment, we used two methods to adjust for the potential confounding effect of age, gender, country of origin, ethnicity, and randomized treatment. The first was the multivariate Cox or competing risk regression model including both grouping and the above variables. The second was assigning weights to each individual according to their propensity score to be obese using standardized mortality ratio weighting [21, 22]. The rationale of the weighting process was to ensure that the weighted estimates of the abovementioned characteristic were comparable among the three groups, and therefore, the corresponding weighted regression model should be free from the confounding effects of these variables.

Spironolactone treatment was shown to have a differential effect on the different subgroups of patients with HFpEF [23]. A sensitivity analysis restricted to the placebo arm was performed using the multivariate regression model.

Missing data were processed as follows. Only a small proportion of patients were excluded (*n* = 9) because of a lack of BMI information as mentioned before. For LCA model, missing data were also input into the algorithm in R. Baseline characteristics were estimated after excluding individuals with missing data.

All P-values are two-sided and *P* < 0.05 was considered significant. All analyses were performed using Stata version 15 (Stata Corp., College Station, TX, USA) and R.

## Results

**Fig. 1:**
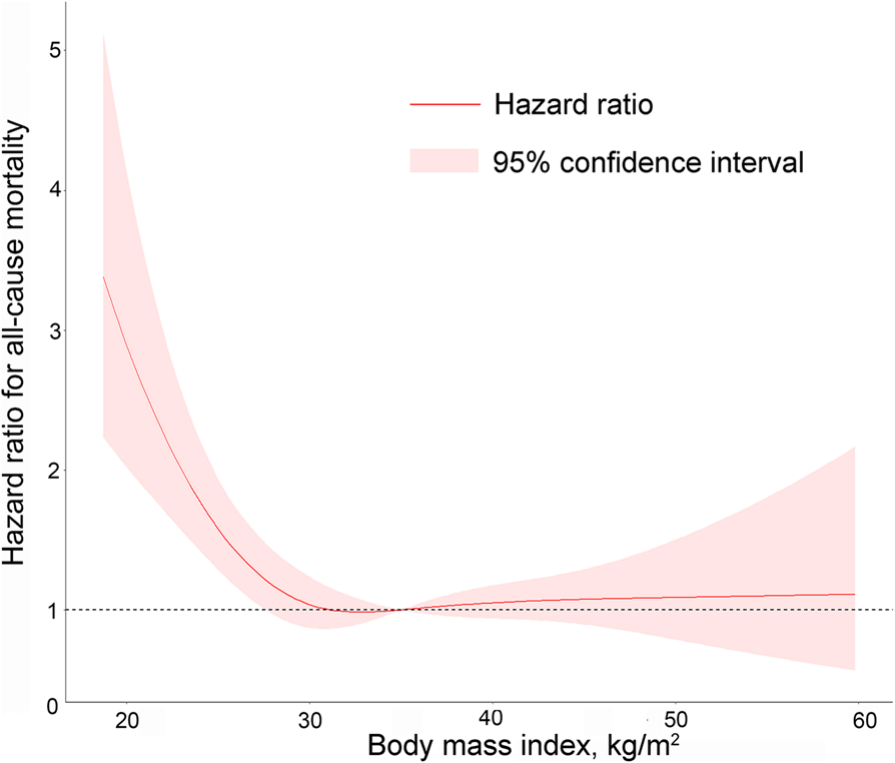
Non-linear association of BMI and risk of all-cause mortality

We first confirmed the existence of the “obesity paradox” in the Americas TOPCAT HFpEF population. The nonlinear relation between BMI and mortality risk was modeled using a univariate Cox proportional hazard model with restrictive cubic splines. The result is shown in Fig. 1. When BMI was lower than 30 kg/m^2^, it was inversely associated with mortality risk. However, when BMI exceeded 30 kg/m^2^, it was no longer associated with mortality risk.

**Fig. 2:**
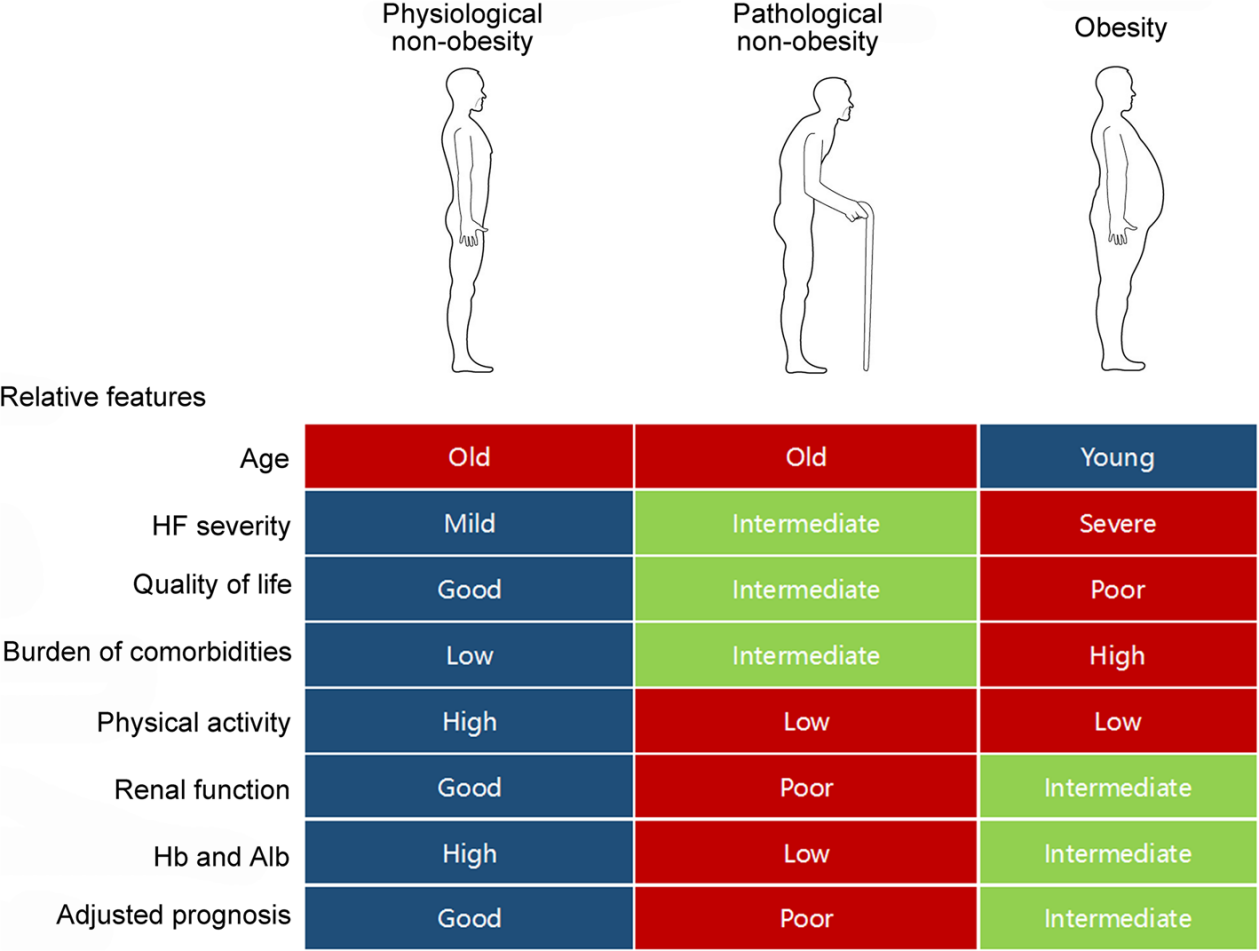
Clinical manifestations and prognosis of the group of physiological non-obesity, pathological non-obesity, and obesity. HFpEF, heart failure with preserved ejection fraction

Among the 623 nonobese patients with HFpEF, the LCA model identified two distinct subgroups, which we named the physiological non-obesity and the pathological non-obesity. Physiological non-obesity accounted for 45.7% (285 out of 623) of the nonobese population, while the pathological non-obesity accounted for the remaining 54.3% (338 out of 623). To better understand the clinical implications of these two nonobese subgroups, 1135 obese (BMI ≥ 30 kg/m^2^) patients were also included in the comparison. Features of these three patient groups were summarized in Fig. 2.

### Baseline characteristics of the nonobese subgroups and obese group

The baseline characteristics of the groups were summarized in Table 1.

**Table 1:** Baseline characteristic of the 3 groups^a^

Characteristic	Physiological non-obesity (*N*=285)	Pathological non-obesity (*N*=338)	Obesity (*N*=1135)	*P*
**LCA variables**				
White race, *n* (%)	246/285 (86.32)	266/338 (78.70)*	865/1135 (76.21)*	0.001
KCCQ overall score	75.00 (56.25, 86.98)	62.24 (42.71, 76.56)*	54.69 (35.68, 73.18)*#	<0.001
Previous HF hospitalization within 12 months, *n* (%)	111/284 (39.08)	200/338 (59.17)*	724/1135 (63.79)*	<0.001
DM, *n* (%)		*	*#	<0.001
DM without insulin usage	34/284 (11.97)	80/338 (23.67)	294/1135 (25.90)	
DM with insulin usage	5/284 (1.76)	59/338 (17.46)	314/1135 (27.67)	
DBP, mmHg	75 (68, 80)	66 (59, 74)*	71 (62, 80)*#	<0.001
Diuretic, *n* (%)	219/285 (76.84)	301/338 (89.05)*	1045/1134 (92.15)*	<0.001
Beta-blocker, *n* (%)	214/285 (75.09)	273/338 (80.77)	893/1134 (78.75)	0.22
GFR, ml/min*1.73m^2^	68.46 (56.56, 80.71)	55.28 (44.09, 69.79)*	60.86 (48.79, 76.95)*#	<0.001
Hb, g/dL	14.09±1.26	11.86±1.32*	12.84±1.65*#	<0.001
Alb, mg/L	4.1 (3.9, 4.4)	3.8 (3.5, 4)*	3.9 (3.6, 4.2)*#	<0.001
**Demographics**				
Age, years	76 (68, 81)	78 (72, 83)*	69 (62, 77)*#	<0.001
Male, *n* (%)	163/285 (57.19)	160/338 (47.34)*	557/1135 (49.07)*	0.03
Hispanic, Latino, or Spanish origin, *n* (%)	68/285 (23.86)	58/338 (17.16)	189/1135 (16.65)*	0.02
Country, *n* (%)		*	*#	<0.001
USA	169/285 (59.30)	202/338 (59.76)	776/1135 (68.37)	
Canada	45/285 (15.79)	89/338 (26.33)	192/1135 (16.92)	
Brazil	41/285 (14.39)	33/338 (9.76)	88/1135 (7.75)	
Argentina	30/285 (30.53)	14/338 (4.14)	79/1135 (6.96)	
Randomized spironolactone treatment	142/285 (49.82)	177/338 (52.37)	555/1135 (48.90)	0.534
**HF related variables**				
NYHA III/IV, *n* (%)	68/285 (23.86)	105/338 (31.07)	445/1132 (39.31)*#	<0.001
Nocturnal paroxysmal dyspnea, *n* (%)	22/282 (7.80)	40/333 (12.01)	191/1110 (17.21)*	<0.001
Orthopnea, *n* (%)	50/282 (17.73)	104/337 (30.86)*	392/1119 (35.03)*	<0.001
Rales, *n* (%)	49/280 (17.50)	62/333 (18.62)	180/1110 (16.22)	0.57
JVP≥10 cmH_2_O, *n* (%)	46/271 (16.97)	64/317 (20.19)	192/1066 (18.01)	0.57
Edema, *n* (%)	179/284 (63.03)	209/338 (61.83)	871/1135 (76.74)*#	<0.001
EF, %	57.0 (50, 61)	59.5 (53, 65)	59.0 (54, 65)	0.07
**Physical examination**				
BMI, kg/m^2^	26.85 (24.69, 28.50)	26.08 (23.46, 27.99)	36.57 (33.08, 41.42)*#	<0.001
HR, bpm	66 (60, 74)	66 (60, 75)	69 (62, 76)*#	<0.001
SBP, mmHg	127 (117, 138)	124 (114, 136)	130 (118, 140)#	<0.001
**Comorbidities**				
CHD, *n* (%)				0.55
CHD without MI	79/284 (27.82)	96/338 (28.40)	280/1135 (24.67)	
CHD with MI	56/284 (19.72)	71/338 (21.01)	231/1135 (20.35)	
Stroke, *n* (%)	21/284 (7.39)	28/338 (8.28)	109/1135 (9.60)	0.45
COPD, *n* (%)	43/284 (15.14)	51/338 (15.09)	197/1135 (17.36)	0.48
Asthma, *n* (%)	24/284 (8.45)	24/338 (7.10)	146/1135 (12.86)#	0.004
Hypertension, *n* (%)	242/284 (85.21)	292/338 (86.39)	1047/1135 (92.25)*#	<0.001
PAD, *n* (%)	27/284 (9.51)	35/338 (10.36)	142/1135 (12.51)	0.27
Dyslipidemia, *n* (%)	185/284 (65.14)	225/338 (66.57)	837/1135 (73.74)*#	0.002
Pacemaker, *n* (%)	44/284 (15.49)	66/338 (19.53)	132/1135 (11.63)#	0.001
AF, *n* (%)	126/284 (44.37)	157/338 (46.45)	458/1135 (40.35)	0.10
Smoking, *n* (%)				0.04
Former smoker	139/284 (48.94)	166/338 (49.11)	592/1134 (52.20)	
Current smoker	30/284 (10.56)	19/338 (5.62)	67/1134 (5.91)	
METs per week^b^	4.00 (0.67, 10.00)	2.33 (0, 7.00)*	1.50 (0, 7.00)*	<0.001
**Medication usage**				
ACEI/ARB, *n* (%)	193/285 (67.72)	258/338 (76.33)	936/1134 (82.54)*#	<0.001
Nitrate, *n* (%)	33/285 (11.58)	67/338 (19.82)*	203/1134 (17.90)*	0.02
**Laboratory results**				
BNP, pg/ml	239.5 (146, 442)	331.5 (203, 626)*	239 (143, 402)#	<0.001
NT-pro BNP, pg/ml	831 (501, 1462)	1740.5 (792.5, 3252)*	884.5 (500, 1610.5)#	<0.001

^a^Baseline characteristics were evaluated after excluding all individuals with missing values

*p<0.05 compared with physiological non-obesity. #*p*<0.05 compared with pathological non-obesity

^b^Calculation of METs per week was based on self-reported frequency and duration of heavy, medium, and light activities

*ACEI*, angiotensin-converting enzyme inhibitor; *AF*, atrial fibrillation; *Alb*, albumin; *ARB*, angiotensin-receptor blocker; *BMI*, body mass index; *CHD*, coronary heart disease; *COPD*, chronic obstructive pulmonary disease; *DBP*, diastolic blood pressure; *DM*, diabetes mellitus; *EF*, ejection fraction; *GFR*, glomerular filtration rate; *Hb*, hemoglobin; *HF*, heart failure; *HR*, heart rate; *JVP*, jugular venous pressure; *KCCQ*, Kansas City Cardiomyopathy Questionnaire; *MI*, myocardial infarction; *NYHA*, New York Heart Association; *PAD*, peripheral arterial disease; *SBP*, systolic blood pressure

The patients with physiological non-obesity had a median age of 76, a male proportion of 57.19%, and a median BMI of 26.85 kg/m^2^. Only 23.86% of the patients were classified as NYHA III/IV. The median KCCQ overall score for quality of life was 75. For metabolic comorbidities, 85.21% had hypertension, 65.14% had dyslipidemia, and 13.73% had diabetes. These patients were relatively active with a median of 4 metabolic equivalents of task (METs) per week. Approximately 76.84% of patients relied on diuretics to relieve HF syndromes. Median GFR was 68.46 ml/min/1.73m^2^. For the nutritional indexes, the mean hemoglobin concentration was 14.09 g/dL and the median serum albumin concentration was 4.1mg/L.

The median age of the patients with pathological non-obesity was 78, and 47.34% were male. The median BMI was 26.08, which was comparable to the Physiological non-obesity group. Approximately 31.07% of them were classified as NYHA III/IV. The KCCQ overall score was lower than patients with physiological non-obesity, with a median of 62.24. The prevalence of hypertension (86.39%) and dyslipidemia (66.57%) were comparable to the Physiological non-obesity group, but the prevalence of diabetes was much higher (41.13%). The patients were less active with a median of 2.3 METs per week. A higher proportion of them relied on diuretics for HF (89.05%). Renal function was worse with a median GFR of 55.28 mL/min/1.73 m^2^. The hemoglobin (average 11.86 g/dL) and serum albumin (median 3.8 mg/L) concentrations were also lower than patients with physiological non-obesity.

**Fig. 3:**
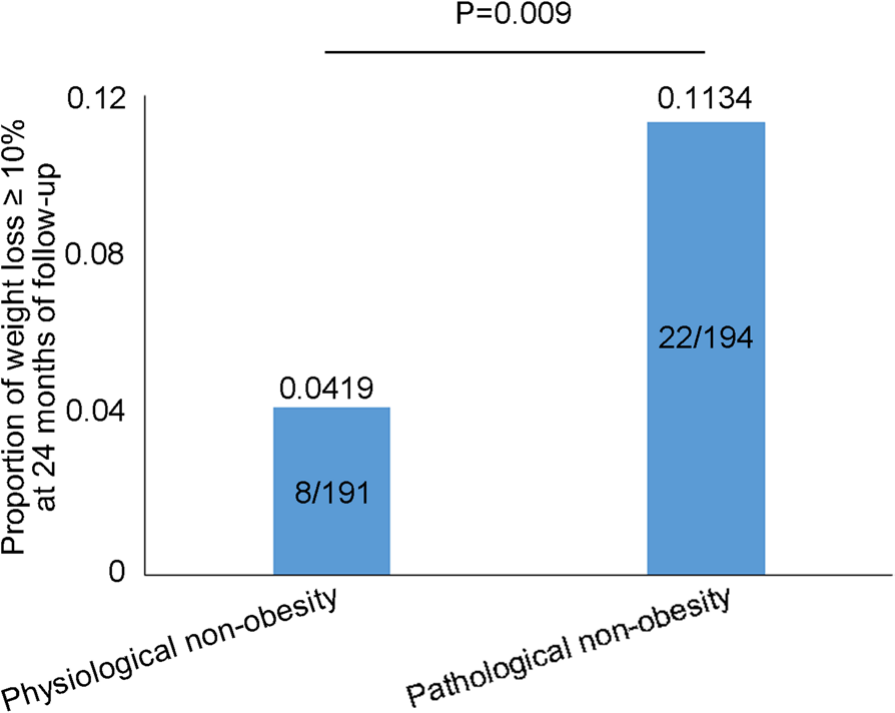
Proportion of patients losing weight of ≥ 10% baseline body weight after 24 months of follow-up in 2 nonobese subgroups

After 2 years of follow-up, patients with pathological non-obesity were more likely to experience weight loss of ≥10% body weight compared to patients with physiological non-obesity (11.34% vs. 4.19%, *P* = 0.009) (Fig. 3).

Patients with obesity were relatively younger with a median age of 69, and 49.07% of them were males. The median BMI (36.57 kg/m^2^) was high. Approximately 39.31% of this group was classified as NYHA III/IV, which was higher than that of both nonobese groups. The quality of life was worse than that in the nonobese groups with a median KCCQ overall score of 54.69. Metabolic syndromes were the most prevalent, with 92.25% of hypertension, 73.74% of dyslipidemia, and 53.57% of diabetes. The patients were also less active compared to patients with physiological non-obesity with a median of 1.5 METs per week; 92.15% of them relied on diuretics for treatment. GFR, hemoglobin concentration, and serum albumin concentration lay between the two nonobese groups (median GFR = 60.86 ml/min/1.73m^2^, mean hemoglobin = 12.84 g/dL, median albumin = 3.9 mg/L).

### Association of nonobese subgroups with clinical outcomes

**Fig. 4:**
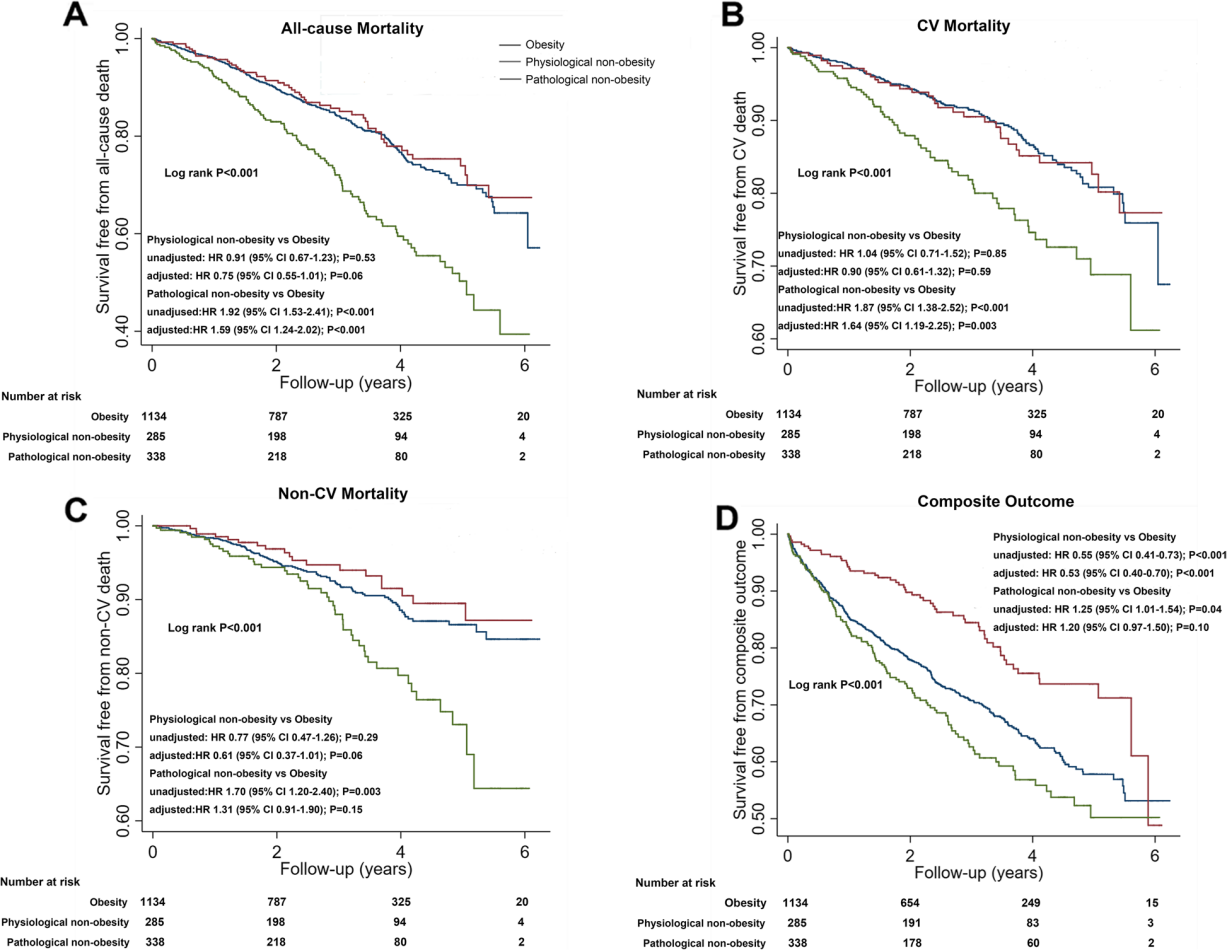
Kaplan-Meier survival curves of the group of physiological non-obesity, pathological non-obesity, and obesity in terms of **A** all-cause mortality, **B** cardiovascular (CV) mortality, **C** noncardiovascular (non-CV) mortality, and **D** composite outcome. CI, confidence interval; CV, cardiovascular; non-CV, noncardiovascular; HR, hazard ratio

For the overall cohort, 383 (21.8%) patients died, 222 of them died of cardiovascular causes, and 161 of them died of noncardiovascular causes. Five hundred and nineteen (29.5%) patients experienced a composite outcome. The crude rates of these outcomes were compared among the 3 groups. Patients with pathological non-obesity had the highest all-cause, cardiovascular, and noncardiovascular mortality rates among the three groups. Patients with physiological non-obesity and obesity had comparable mortality rates, but the patients with physiological non-obesity had a lower rate of composite outcome (Table 2). The Kaplan-Meier survival curve and the unadjusted regression model showed similar trends (Fig. 4).

**Table 2:** Crude rate of outcomes in the 3 groups

	Physiological non-obesity (*N*=285)	Pathological non-obesity (*N*=338)	Obesity (*N*=1135)	*P*
All-cause death, *n* (%)	52 (18.25)	112 (33.14)*	219 (19.30)#	<0.001
CV death, *n* (%)	33 (11.58)	65 (19.23)*	124 (10.93)#	<0.001
Non-CV death, *n* (%)	19 (6.67)	47 (13.91)*	95 (8.37)#	0.002
Composite outcome, *n* (%)	54 (18.95)	119 (35.21)*	346 (30.48)*	<0.001

*p<0.05 compared with physiological non-obesity. #*p*<0.05 compared with pathological non-obesity.

*CV*, cardiovascular

To adjust for the potential confounding effects of the demographic differences in age, gender, country of origin, ethnicity, and randomized treatment, the multivariate regression, and regression with standardized mortality ratio weighting were performed.

In the multivariate regression model, compared to patients with obesity, patients with physiological non-obesity had a 47% decrease in risk of HF composite outcome (HR 0.53, 95% CI 0.40–0.70, *P* < 0.001) and a trend of lower all-cause mortality risk (HR 0.75, 95% CI 0.55–1.01, *P* = 0.06). In contrast, patients with pathological non-obesity were associated with a 59% increase in all-cause mortality risk (HR 1.59, 95% CI 1.24–2.02, *P* < 0.001), which appeared to be driven by an increased cardiovascular mortality risk (HR 1.64, 95% CI 1.19–2.25, *P* = 0.003) (Fig. 4).

**Fig. 5:**
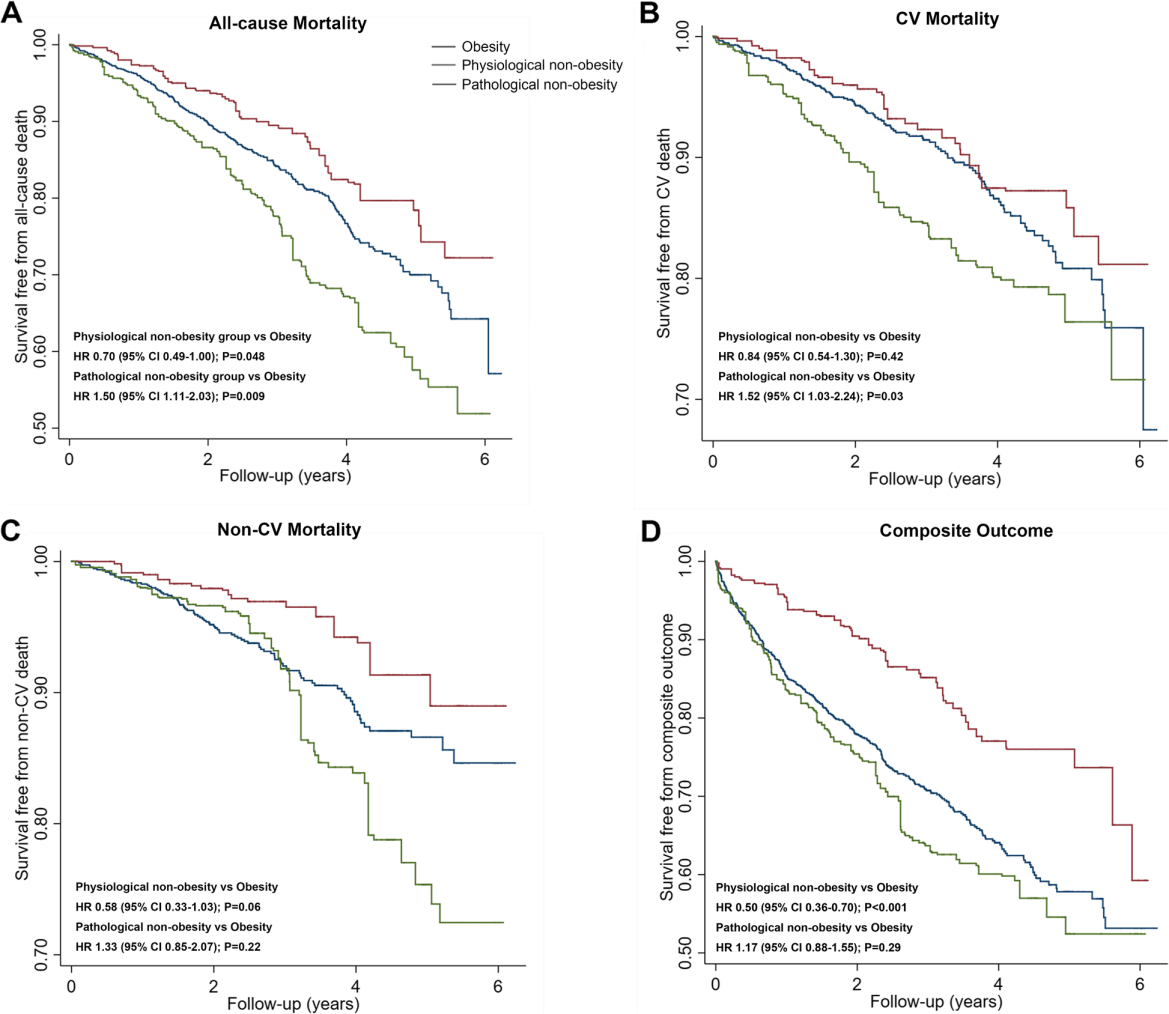
Weighted Kaplan-Meier survival curves of the group of physiological non-obesity, pathological non-obesity, and obesity in terms of **A** all-cause mortality, **B** cardiovascular (CV) mortality, **C** noncardiovascular (non-CV) mortality, and **D** composite outcome. CI, confidence interval; CV, cardiovascular; non-CV, noncardiovascular; HR, hazard ratio

Using standardized mortality ratio weighted models yielded similar results. Age, gender, country of origin, ethnicity, and randomized treatment were well balanced after weighting (Additional file 1: Table S6). Patients with physiological non-obesity were associated with a lower risk of all-cause mortality (HR 0.70, 95% CI 0.49–1.00, *P*=0.048) and HF composite outcome (HR 0.50, 95% CI 0.36–0.70, *P*<0.001), while patients with pathological non-obesity were associated with a higher risk of all-cause (HR 1.50, 95% CI 1.11–2.03, *P*=0.009) and cardiovascular (HR 1.52, 95% CI 1.03–2.24, *P*=0.03) mortality (Fig. 5).

The sensitivity analysis restricted the multivariate models in the placebo arm and yielded similar results to the analysis in the overall population (Table 3), except that some differences were no longer significant due to a reduced sample size.

**Table 3:** Association of nonobese subgroups with clinical outcomes in a sensitivity analysis that only included the placebo arm

	Unadjusted		Adjusted	
	HR (95% CI)	*p*	HR (95% CI)	*p*
All-cause mortality				
Obesity	Reference		Reference	
Physiological non-obesity	0.91 (0.60–1.38)	0.66	0.74 (0.48–1.14)	0.18
Pathological non-obesity	1.97 (1.44–2.69)	<0.001	1.53 (1.10–2.13)	0.01
Cardiovascular mortality				
Obesity	Reference		Reference	
Physiological non-obesity	1.13 (0.69–1.86)	0.64	0.97 (0.59–1.59)	0.90
Pathological non-obesity	1.75 (1.17–2.62)	0.006	1.40 (0.92–2.13)	0.12
Non-cardiovascular mortality				
Obesity	Reference		Reference	
Physiological non-obesity	0.60 (0.27–1.33)	0.21	0.48 (0.21–1.07)	0.07
Pathological non-obesity	1.93 (1.18–3.16)	0.008	1.48 (0.87–2.52)	0.15
Composite outcome				
Obesity	Reference		Reference	
Physiological non-obesity	0.56 (0.38–0.82)	0.003	0.55 (0.37–0.81)	0.003
Pathological non-obesity	1.15 (0.87–1.54)	0.33	1.14 (0.84–1.54)	0.41

*CI*, confidence interval; *HR*, hazard ratio

## Discussion

In this study, we identified two subgroups of nonobese patients with HFpEF using LCA. The differences in baseline characteristics, long-term weight change, and prognoses indicate that the implication of low BMI in the two nonobese groups was distinct. In the physiological non-obesity group, the low BMI might represent a condition free from obesity-related damages, while that in the Pathological non-obesity group represented a diseased condition that is associated with poor prognosis. Our analysis emphasized the heterogeneity of nonobese patients with HFpEF. Pooling different subgroups of the nonobese HFpEF together might be the reason for the “obesity paradox” in HFpEF.

Obesity is a fundamental pathogenic factor for HFpEF and obese HFpEF is considered a subphenotype of HFpEF with distinct cardiac structures and hemodynamics compared with the nonobese controls [7, 8]. However, numerous studies have indicated that overweight or obese patients with HFpEF had a better prognosis compared with their leaner counterparts, the so-called obesity paradox. A study involving 2501 ambulatory HFpEF showed the highest mortality rate in patients with normal weight [24]. A post hoc analysis of the overall TOPCAT population also indicated that overweight and obesity were both associated with a lower mortality risk [25]. We also confirmed the existence of the “obesity paradox” in our analyzed population.

The heterogeneity of both the obese and nonobese patients contributed to the “obesity paradox.” Most of the published studies focused on the heterogeneity of obese patients. BMI is still the most widely used measurement to define obesity. However, BMI was not an ideal measurement for body composition and adipose distribution. Indeed, BMI only moderately correlated with exercise tolerance among patients with HF [26], but skeletal muscle composition and different patterns of regional adipose distribution were more closely related to exercise capability [27, 28]. Specifically, epicardial adipose tissue was associated with hemodynamics, metabolic profile, and survival among patients with HF [29]. As BMI only captures the overall body weight, adiposity deposition in BMI-defined obese patients could be heterogeneous. From the perspective of nonobese patients, HF is a chronic wasting disease and cardiac cachexia could cause unintentional weight loss. Therefore, a part of the “normal BMI” in HF could be pathological, which has opposite clinical implications to physiological normal weight. Pooling the patients with pathological and physiological non-obesity together could be one of the reasons for “obesity paradox.” Using the LCA clustering technique, we identified two nonobese HFpEF subgroups with distinct prognostic implications. Interestingly, the Pathological non-obesity group had the lowest serum albumin and hemoglobin, which suggested patients in this group might suffer from cardiac cachexia [30]. A higher proportion of significant weight loss also supported the existence of cachexia. These results provided a novel explanation for the “obesity paradox” in a data-driven way.

The distinct features of the three HFpEF groups indicate different natural histories of HFpEF. The relatively younger baseline age in the group of obesity suggests the early onset of HFpEF among these patients, which could be explained by the severe metabolic syndrome in these patients because they also had the most prevalent hypertension, hyperlipidemia, and diabetes. These metabolic disorders are believed to contribute to a systematic pro-inflammatory state and development of HFpEF [31]. The patients with pathological non-obesity also had a relatively heavy burden of comorbidities. Specifically, the high prevalence of diabetes did not match their relatively low BMI. Interestingly, demographic features like gender, race, and ethnicity were comparable between patients with pathological non-obesity and obesity, suggesting that these two groups might be the same group of HFpEF at different stages. We hypothesized that the patients with pathological non-obesity used to be obese when they were young, but they gradually lost their weight unintentionally due to cardiac cachexia as HFpEF progressed and became nonobese at an older age. The poor prognosis of this group also fits in with this hypothesis. Interestingly, there was a mismatch between the comparative quality of life, HF manifestation, and prognosis when comparing the obesity group with the pathological non-obesity group. Patients with pathological non-obesity had a better quality of life, less severe HF symptoms, but a worse prognosis. Such mismatch was also seen in other studies on HFpEF [32]. This mismatch could be explained by the difference in the quality of life and symptoms between younger and older patients with HF. Previous studies found that younger patients with HF tended to have poorer quality of life [33] and more severe symptoms of edema [34] or dyspnea [35]. Comorbidities were the least prevalent in the Physiological non-obesity group. However, the absolute prevalence of HFpEF risk factors was actually rather high. In this subgroup, 47.54% suffered from coronary heart disease, 85.21% suffered from hypertension, 44.37% suffered from atrial fibrillation, 13.7% suffered from diabetes, and 33.8% suffered from chronic kidney disease. Besides, this subgroup was old with a median age of 76 years. HFpEF had overlapping characteristics with aging-related cardiac dysfunction, and aging was considered an important pathogenesis factor of HFpEF [36]. Therefore, aging might play a larger part in the pathogenesis of HFpEF in this subgroup compared with the other 2 subgroups. This was consistent with their high quality of life, mild HF symptoms, high level of weekly physical activity, and good prognosis.

Our results further emphasized that the “obesity paradox” did not mean a beneficial effect of adiposity in HFpEF. Instead, the poor prognosis of the nonobese group was driven by a pathological subgroup. The “true” subgroup of nonobese HFpEF had a better prognosis compared with the obese patients with HFpEF. Two studies [14, 23], including one from our group [23], used LCA to analyze the whole HFpEF population in the TOPCAT dataset. Both studies identified 3 HFpEF subgroups. These studies were designed to explore the heterogeneity of the HFpEF population and the potential differential treatment effect of the medication but not to explain the “obesity paradox” in patients with HFpEF. Although the prevalence of obesity was different among those three subgroups, the absolute percentage of obese patients was at least 44% [23]. Therefore, the comparison of the subgroups generated from these studies could not provide any insight into the unsolved question of the “obesity paradox.” In contrast, the present study aimed to explain the paradox by resolving the heterogeneity of the nonobese patients. With a different design, the subgroups identified in the present study was different from those generated by previous studies in baseline characteristics, and as a result, prognosis. The present findings suggested that the poor prognosis of nonobese patients with HFpEF was driven by a subgroup with pathological non-obesity, which added to the understanding of “obesity paradox.”

### Limitations and strengths

Several limitations should be taken into consideration. First, our analysis was restricted to HFpEF. It is not clear whether these findings could be generalized to HFrEF. Second, this was a hypothesis-generating study without external validation. Our findings need to be validated by future studies. Third, the sample size, especially for the nonobese group, was limited. Fourth, the history of obesity and body weight change before trial recruitment could provide more useful insights, but this information was not available. Fifth, the cachexic nature of patients with pathological non-obesity was inferred from the clinical manifestation and prognosis. A more comprehensive nutritional evaluation or assessment of the adipose tissue and skeletal muscle mass was not available to confirm the existence of cachexia. Sixth, the difference biological effect of visceral adipose tissue and subcutaneous adipose tissue might contribute to the obesity paradox. However, this information was not available in the TOPCAT dataset. The fat distribution in different subgroups of nonobese HFpEF needed to be evaluated in future studies. Seventh, the present analysis mainly focused on nonobese patients to explain the “obesity paradox.” However, obese patients with HFpEF could also be heterogeneous as BMI did not reflect adipose distribution in the body.

There were also strengths in this study. First, the LCA model comprehensively assessed multiple features of the nonobese HFpEF population, which was better in terms of subgrouping compared with the traditional subgroup analysis based on a single variable. In addition, the use of an algorithm-based LCA variable selection further reduced the subjective bias in subgrouping the nonobese patients with HFpEF. Furthermore, the prognostic difference was confirmed using separate analysis with different methods to adjust for potential demographic confounders and a sensitivity analysis with only the placebo arm.

## Conclusions

In this study, two distinct subgroups of nonobese patients with HFpEF were identified using LCA. One subgroup had a relatively “healthy” baseline clinical profile, while the other had more comorbidities and low levels of albumin and hemoglobin. Importantly, these two subgroups had opposite prognostic implications compared with obese patients with HFpEF, which indicates the different clinical significance of low BMI in 2 subgroups. The low BMI was likely physiological in the former subgroup, but pathological resulting from unintentional weight loss in the latter subgroup. Our study provides evidence for the heterogeneity among the nonobese patients with HFpEF and an alternative explanation for the “obesity paradox” in a data-driven manner.

## Supplementary Information

**Additional file 1: Table S1.** A list of manually selected variables. **Table S2**. Process of algorithm-based variable selection. **Table S3.** Coding of selected variables for LCA. **Table S4.** Goodness-of-fit statistics of 2-5 classes. **Table S5.** Partial probabilities of subgroup membership for selected variables. **Table S6.** Weighted estimated of demographic features and treatment arm of 3 groups.

## Data Availability

Data of the TOPCAT trial were obtained from the National Institutes of Heart, Lung, and Blood Institute’s Biologic Specimen and Data Repository Information Coordinating Center via an approved proposal. The use of data was in accordance with the terms from the center.

## Abbreviations

aBIC: Adjusted BIC
ACEI: Angiotensin-converting enzyme inhibitor
AIC: Akaike information criterion
ARB: Angiotensin-receptor blocker
BMI: Body mass index
BIC: Bayesian Information Criterion
cAIC: Consistent AIC
CI: Confidence interval
CV: Cardiovascular
GFR: Glomerular filtration rate
HF: Heart failure
HFpEF: Heart failure with preserved ejection fraction
HR: Hazard ratio
IQR: Interquartile range
KCCQ: Kansas City Cardiomyopathy Questionnaire
NYHA: New York Heart Association
LCA: Latent class analysis
SD: Standard deviation
TOPCAT: Treatment of Preserved Cardiac Function Heart Failure with an Aldosterone Antagonist Trial
