# Supplementary material for: Distinct implications of body mass index in different subgroups of nonobese patients with heart failure with preserved ejection fraction: a latent class analysis of data from the TOPCAT trial
Source: BMC Med. 2022 Nov 2;20:423. doi: 10.1186/s12916-022-02626-4 (PMC9632105; doi:10.1186/s12916-022-02626-4)
Supplement: Supplementary file 1 — Additional file 1: Table S1. A list of manually selected variables. Table S2. Process of algorithm-based variable selection. Table S3. Coding of selected variables for LCA. Table S4. Goodness-of-fit statistics of 2-5 classes. Table S5. Partial probabilities of subgroup membership for selected variables. Table S6. Weighted estimated of demographic features and treatment arm of 3 groups. [file 12916_2022_2626_MOESM1_ESM.docx]

Table S1 A list of manually selected variables

| Demographics | HF related variables | Quality of life | Physical examination | Comorbidities | Medication usage | Laboratory results | Life style |
| --- | --- | --- | --- | --- | --- | --- | --- |
| Race | NYHA class | KCCQ overall score | HR | CHD | Diuretic | Na | Smoking |
| Age | Edema |  | SBP | COPD | Beta-blocker | Hb | Alcohol |
| Gender | EF |  | DBP | Hypertension | CCB | Alb | Mets per week |
|  | Previous HF hospitalization |  |  | PAD | ACEI/ARB | Cr | Salt intake |
|  |  |  |  | Dyslipidemia | Statin | K |  |
|  |  |  |  | Thyroid disease | Antidepressant | GFR |  |
|  |  |  |  | DM |  | HCT |  |
|  |  |  |  | AF |  |  |  |

ACEI, angiotensin-converting enzyme inhibitor; AF, atrial fibrillation; Alb, albumin; ARB, angiotensin-receptor blocker; BMI, body mass index; CCB, calcium channel blockers; CHD, coronary heart disease; COPD, chronic obstructive pulmonary disease; Cr, creatinine; DBP, diastolic blood pressure; DM, diabetes mellitus; EF, ejection fraction; GFR, glomerular filtration rate; Hb, hemoglobin; HCT, hematocrit; HF, heart failure; HR, heart rate; JVP, jugular venous pressure; KCCQ, Kansas city cardiomyopathy questionnaire; MI, myocardial infarction; NYHA, New York Heart Association; PAD, peripheral arterial disease; SBP, systolic blood pressure.

Table S2 Process of algorithm-based variable selection

| Step | Variable | BIC diff | Decision |
| --- | --- | --- | --- |
| Remove | GFR | 836.43 | Accepted |
| Remove | HCT | 663.50 | Accepted |
| Add | GFR | -767.87 | Rejected |
| Remove | Gender | 140.96 | Accepted |
| Add | GFR | -477.78 | Rejected |
| Remove | SBP | 101.25 | Accepted |
| Add | Gender | -141.04 | Rejected |
| Remove | Age | 40.29 | Accepted |
| Add | SBP | -101.14 | Rejected |
| Remove | Hypertension | 40.42 | Accepted |
| Add | Age | -40.96 | Rejected |
| Remove | Dyslipidemia | 35.28 | Accepted |
| Add | Age | -35.98 | Rejected |
| Remove | Statin | 33.56 | Accepted |
| Add | Gender | -33.00 | Rejected |
| Remove | AF | 25.62 | Accepted |
| Add | Age | -22.89 | Rejected |
| Remove | Smoke | 24.42 | Accepted |
| Add | Age | -25.36 | Rejected |
| Remove | Alcohol | 21.28 | Accepted |
| Add | Age | -24.17 | Rejected |
| Remove | HR | 17.38 | Accepted |
| Add | Alcohol | -21.84 | Rejected |
| Remove | EF | 16.90 | Accepted |
| Add | HR | -17.52 | Rejected |
| Remove | Salt intake | 14.75 | Accepted |
| Add | EF | -16.24 | Rejected |
| Remove | Na | 12.43 | Accepted |
| Add | Salt intake | -14.88 | Rejected |
| Remove | K | 11.71 | Accepted |
| Add | Na | -11.72 | Rejected |
| Remove | NYHA class | 12.14 | Accepted |
| Add | Na | -11.55 | Rejected |
| Remove | CHD | 12.00 | Accepted |
| Add | Na | -11.91 | Rejected |
| Remove | Edema | 10.55 | Accepted |
| Add | Na | -11.95 | Rejected |
| Remove | COPD | 9.92 | Accepted |
| Add | Edema | -10.57 | Rejected |
| Remove | Mets per week | 9.15 | Accepted |
| Add | COPD | -10.12 | Rejected |
| Remove | Thyroid disease | 8.91 | Accepted |
| Add | Mets per week | -9.11 | Rejected |
| Remove | ACEI/ARB | 8.30 | Accepted |
| Add | Thyroid disease | -8.84 | Rejected |
| Remove | CCB | 5.35 | Accepted |
| Add | Hypertension | -6.08 | Rejected |
| Remove | PAD | 4.14 | Accepted |
| Add | CCB | -4.93 | Rejected |
| Remove | Cr | 4.96 | Accepted |
| Add | GFR | 7.77 | Accepted |
| Remove | Antidepressant | 1577.70 | Accepted |
| Add | CCB | -1576.90 | Rejected |
| Remove | Beta blocker | -1570.44 | Rejected |
| Add | CCB | -1576.90 | Rejected |

ACEI, angiotensin-converting enzyme inhibitor; AF, atrial fibrillation; Alb, albumin; ARB, angiotensin-receptor blocker; BMI, body mass index; CCB, calcium channel blockers; CHD, coronary heart disease; COPD, chronic obstructive pulmonary disease; Cr, creatinine; DBP, diastolic blood pressure; DM, diabetes mellitus; EF, ejection fraction; GFR, glomerular filtration rate; Hb, hemoglobin; HCT, hematocrit; HF, heart failure; HR, heart rate; JVP, jugular venous pressure; KCCQ, Kansas city cardiomyopathy questionnaire; MI, myocardial infarction; NYHA, New York Heart Association; PAD, peripheral arterial disease; SBP, systolic blood pressure.

| Table S3 Coding of selected variables for LCA | | | | |
| --- | --- | --- | --- | --- |
| Variables | Coding | | | |
|  | 1 | 2 | 3 | 4 |
| Race | White | Other |  |  |
| Previous HF hospitalization | No | Yes |  |  |
| DM | None | DM without insulin usage | DM with insulin usage |  |
| KCCQ overall score | <45 | 45-65 | 65-85 | ≥85 |
| DBP, mmHg | <60 | 60-70 | 70-80 | ≥80 |
| Diuretic | No | Yes |  |  |
| Beta-blocker | No | Yes |  |  |
| Hb, g/dL | <12 | 12-13 | 13-14 | ≥14 |
| Alb, g/dL | <3.6 | 3.6-3.9 | 3.9-4.2 | ≥4.2 |
| GFR, ml/min/1.73m^2^ | <50 | 50-60 | 60-70 | ≥70 |
| Alb, albumin; DBP, diastolic blood pressure; DM, diabetes mellitus; GFR, glomerular filtration rate; Hb, hemoglobin; HF, heart failure; KCCQ, Kansas city cardiomyopathy questionnaire. | | | | |

| Table S4 Goodness-of-fit statistics of 2-5 classes |
| --- |

| Number of classes | BIC | AIC | X^2^ | G^2^ | cAIC | aBIC |
| --- | --- | --- | --- | --- | --- | --- |
| 2 | 12118.59 | 11927.9 | 46039.18 | 3821.301 | 12161.59 | 11982.07 |
| 3 | 12189.46 | 11901.21 | 46462.37 | 3758.553 | 12254.46 | 11983.09 |
| 4 | 12273.35 | 11887.54 | 38718.7 | 3702.646 | 12360.35 | 11997.14 |
| 5 | 12371.29 | 11887.92 | 39621.81 | 3662.248 | 12480.29 | 12025.23 |

AIC, Akaike information criterion; aBIC, adjusted Bayesian information criterion; cAIC，consistent AIC.

Table S5 Partial probabilities of subgroup membership for selected variables

| Variables | | Physiologically nonobese group | | Pathologically nonobese group | |  |
| --- | --- | --- | --- | --- | --- | --- |
| Race | White | 0.8692 | | 0.7839 | |  |
|  | Other | 0.1308 | | 0.2161 | |  |
| Previous HF Hospitalization | No | 0.5971 | | 0.4225 | |  |
|  | Yes | 0.4029 | | 0.5775 | |  |
| DM | None | 0.8460 | | 0.6084 | |  |
|  | DM without insulin usage | 0.1242 | | 0.2304 | |  |
|  | DM with insulin usage | 0.0297 | | 0.1613 | |  |
| KCCQ overall score | <45 | 0.1390 | | 0.2749 | |  |
|  | 45-65 | 0.2021 | | 0.2444 | |  |
|  | 65-85 | 0.3688 | | 0.3401 | |  |
|  | ≥85 | | 0.2901 | | 0.1406 |  |
| DBP, mmHg | <60 | | 0.0723 | | 0.2404 |  |
|  | 60-70 | | 0.2210 | | 0.3355 |  |
|  | 70-80 | | 0.3687 | | 0.2403 |  |
|  | ≥80 | | 0.3380 | | 0.1838 |  |
| Diuretic | No | | 0.2247 | | 0.1178 |  |
|  | Yes | | 0.7753 | | 0.8822 |  |
| Beta-blocker | No | | 0.2506 | | 0.1925 |  |
|  | Yes | | 0.7494 | | 0.8075 |  |
| Hb, g/dL | <120 | | 0.000 | | 0.512 |  |
|  | 120-130 | | 0.1902 | | 0.2870 |  |
|  | 130-140 | | 0.3439 | | 0.1319 |  |
|  | ≥140 | | 0.4658 | | 0.0692 |  |
| Alb, g/dL | <3.6 | | 0.1002 | | 0.3538 |  |
|  | 3.6-3.9 | | 0.1118 | | 0.2239 |  |
|  | 3.9-4.2 | | 0.4127 | | 0.2715 |  |
|  | ≥4.2 | | 0.3753 | | 0.1508 |  |
| GFR, ml/min/1.73m^2^ | <50 | | 0.1249 | | 0.3595 |  |
|  | 50-60 | | 0.2191 | | 0.2234 |  |
|  | 60-70 | | 0.1958 | | 0.1612 |  |
|  | ≥70 | | 0.4602 | | 0.2559 |  |
| Alb, albumin; DBP, diastolic blood pressure; DM, diabetes mellitus; GFR, glomerular filtration rate; Hb, hemoglobin; HF, heart failure; KCCQ, Kansas city cardiomyopathy questionnaire. | | | | | | |

| Table S6 Weighted estimated of demographic features and treatment arm of 3 groups | | | | |
| --- | --- | --- | --- | --- |
| Characteristic | Physiological  non-obesity  (N=285) | Pathological  non-obesity  (N=338) | Obesity  (N=1135) | P |
| Age, y | 68.97±10.17 | 68.70±9.93 | 69.41±9.35 | 0.66 |
| Male, (%) | 0.48 | 0.43 | 0.49 | 0.31 |
| Hispanic, Latino, or Spanish  origin, (%) | 0.17 | 0.18 | 0.17 | 0.81 |
| Country, (%)  United States  Canada  Brazil  Argentina | 0.68  0.17  0.08  0.08 | 0.67  0.17  0.10  0.07 | 0.68  0.17  0.08  0.07 | 0.95 |
| Spironolactone arm, (%) | 0.48 | 0.50 | 0.51 | 0.76 |
